# Supplementary material for: Web and phone-based COVID-19 syndromic surveillance in Canada: A cross-sectional study
Source: PLoS One. 2020 Oct 2;15(10):e0239886. doi: 10.1371/journal.pone.0239886 (PMC7531838; doi:10.1371/journal.pone.0239886)
Supplement: S1 Table — (DOCX) [file pone.0239886.s001.docx]

**Table 1. Survey Response Rates**

| **Source** | **Response Rate** |
| --- | --- |
| Angus Reid poll | 36.4% |
| COVID Near You | Due to the nature of the participatory surveillance tool, it is not possible to report a response rate. This is because the tool is not sent out but rather accessed by interested members of the public who actively search for or follow a link to the website. |
| Forum poll | First wave was 7.5% and second wave was 4.5% (across both waves, response rate was 5.7%) |
