# Supplementary material for: Web and phone-based COVID-19 syndromic surveillance in Canada: A cross-sectional study
Source: PLoS One. 2020 Oct 2;15(10):e0239886. doi: 10.1371/journal.pone.0239886 (PMC7531838; doi:10.1371/journal.pone.0239886)
Supplement: S2 Table — (DOCX) [file pone.0239886.s002.docx]

**Table 2. Angus Reid poll questions used in this study**

| Let’s think about the last month or so. In that time have you experienced any of the following symptoms? (select all that apply):  - Difficulty breathing / shortness of breath  - a fever  - a mild dry cough  - a severe dry cough (keeps you from sleeping)  - sore throat  - frequent sneezing  - loss of sense of smell  - fever with hallucinations |
| --- |
| When it comes to COVID-19, which of these scenarios best describes your experience (select one)  - I have been tested for COVID-19  - I am scheduled to be tested  - Trying to get tested but haven’t been able to  - Have done a self-assessment through government website/app  - Not tested nor planning to be tested or self assessed |
| Gender  Male  Female  Other |
| Which of the following categories includes your age? (select one)  18-24  25-34  35-44  45-54  55-64  65+ |
| To help us with our analysis, please tell us how many people currently live in your household?  -Just one – I live alone  -Two people  -Three people  -Four people  -Five or more people in my household |
| Which of the following categories best describes your total annual household income before taxes?  Under $25,000  $25,000 to less than $50,000  $50,000 to less than $100,000  $100,000 to less than $150,000  $150,000 to less than $200,000  Over $200,000  Don''t know / Rather not say |
| What is the highest level of education that you yourself completed? (select one)  Some elementary or high school  High school graduate  Some college/trade school  Graduated from college/trade school  Some university  University undergraduate degree, such as a Bachelor’s Degree  University graduate degree, such as a Master’s or PhD |
| **Existing Variables in Dataset** |
| Visible minority  -Yes  - No |
| Province  - British Columbia  - Alberta  - Saskatchewan  - Manitoba  - Ontario  - Quebec  - New Brunswick  - Nova Scotia  - Prince Edward Island  - Newfoundland and Labrador |
| Ethnicity (select up to 15)  - Indigenous/First Nations/Inuit/Metis  - English/Irish/Scottish  - French  - Other European  - Caribbean  - African  - Middle Eastern/Central Asian  - Chinese  - Filipino  - South Asian  - Other Asian  - Oceania  - Other  - Rather not say |
