# Supplementary material for: Web and phone-based COVID-19 syndromic surveillance in Canada: A cross-sectional study
Source: PLoS One. 2020 Oct 2;15(10):e0239886. doi: 10.1371/journal.pone.0239886 (PMC7531838; doi:10.1371/journal.pone.0239886)
Supplement: S3 Table — (DOCX) [file pone.0239886.s003.docx]

**Table 3. COVID Near You tool questions used in this study.**

| **Tool Question** | **Response options** |
| --- | --- |
| How are you feeling? | - Great thanks - Not feeling well |
| What are your symptoms? Select all that apply. | fever, fatigue, runny nose, cough, sneezing, aches and pains, chills/night sweats, sore throat, diarrhea, headache, shortness of breath, nausea, rash, loss of smell/taste, stomach pain/cramps, loss of appetite, other (free text option) |
| What day did you start feeling ill? | Day, month, year |
| Did you see a health professional for these symptoms? | Yes/No |
| Where did you see the health professional? Select all that apply. | Options are: doctor’s office, urgent care centre, in-store clinic, emergency room, hospitalized overnight, virtual visit |
| Have you been tested for COVID-19? | Yes/No |
| What were the results? | - positive – negative - waiting for results |
| Have you traveled outside USA within the 14 days before symptoms onset? | Yes/No |
| To the best of your knowledge, have you been in direct contact with anyone who has a confirmed case of COVID-19? | Yes/No |
| Age | Enter years |
| Gender | - Male – Female - Non-binary/third gender  - Other - Prefer not to say |
| Postal code (first three characters) | 3 characters |
