# Supplementary material for: Web and phone-based COVID-19 syndromic surveillance in Canada: A cross-sectional study
Source: PLoS One. 2020 Oct 2;15(10):e0239886. doi: 10.1371/journal.pone.0239886 (PMC7531838; doi:10.1371/journal.pone.0239886)
Supplement: S4 Table — (DOCX) [file pone.0239886.s004.docx]

**Table 4.** Forum poll questions used in this study

| Q1. Have you, or has anyone in your household had a fever, that is, temperature above 38 degrees Celsius or about 100 degrees Fahrenheit in the past week?  Press 1 for yes  Press 2 for no |
| --- |
| Q2. Are you, or is anyone in your household currently suffering from a new cough in the past week?  Press 1 for yes  Press 2 for no |
| Q3. Are you, or is anyone in your household currently suffering from new headaches in the past week?  Press 1 for yes  Press 2 for no |
| Q4. Are you, or is anyone in your household suffering from a new sore throat in the past week?  Press 1 for yes  Press 2 for no |
| Q5.Are you, or is anyone in your household suffering from a loss of taste or smell in the past week?  Press 1 for yes  Press 2 for no |
| Q6. Are you, or is anyone in your household suffering from new diarrhea in the past week?  Press 1 for yes  Press 2 for no |
| Q7. Are you, or is anyone in your household suffering from a new shortness of breath in the past week?  Press 1 for yes  Press 2 for no |
| Q8. Have you, or has anyone with symptoms in this household been tested for COVID-19 since the onset of symptoms?  Press 1 for yes  Press 2 for no |
| Q9. [[IF Q8=1] Did you, or did anyone in this household test positive for COVID-19?  Press 1 for yes  Press 2 for no |
| Q.10 . [IF Q8=1] Are you, or is anyone in this household still waiting for their COVID-19 test result?  Press 1 for yes  Press 2 for no |
| Q19. The following questions will be used to group our data. What is your gender?  Press 1 for male  Press 2 for female  Press 3 for other or if you don't wish to say |
| Q20. How many people are in your household?  Press 1 for just one person, myself  Press 2 for 2 people  Press 3 for 3 people  Press 4 for 4 people  Press 5 for 5 or more people in the household |
| Q21. How old are you?  Press 1 for Under 24  Press 2 for 25 to 34  Press 3 for 35 to 44  Press 4 for 45 to 54  Press 5 for 55 to 64  Press 6 for 65 to 74  Press 7 for 75 or older |
| Q22 The highest level of education achieved.  Press 1 for secondary school or less  Press 2 for some college or university  Press 3 for completed university  Press 4 for post graduate degree |
| Q23. What is your household's income before taxes?  Press 1 for less than $20,000  Press 2 for $20,000 to $40,000  Press 3 for $40,000 to $60,000  Press 4 for $60,000 to $80,000  Press 5 for $80,000 to $100,000  Press 6 for $100,000 or more  Press 7 if you don't want to say |
