# Supplementary material for: Web and phone-based COVID-19 syndromic surveillance in Canada: A cross-sectional study
Source: PLoS One. 2020 Oct 2;15(10):e0239886. doi: 10.1371/journal.pone.0239886 (PMC7531838; doi:10.1371/journal.pone.0239886)
Supplement: Attachment — Submitted filename: comments_07202020.docx [file pone.0239886.s005.docx]

**Review for PLoS ONE**

**July 17, 2020**

**Title:** **Web and Phone-based COVID-19 Syndromic Surveillance in Canada: A Cross-Sectional Study**

This paper presents a study to describe the characteristics, symptoms, and self-reported testing rates of respondents in three different COVID-19 symptom surveys in Canada. However, there are questions that limit my enthusiasm of the paper, as outlined below.

1. Results:
   a. Table 1: Authors considered (-) and 0. What I guess (-) shows cell<6. So please define that at the caption and fix that across all 3 tables. We don’t expect to have both 0 and (-) across tables.
   b. Table 2: Authors did stratification for the age based on the gender.
      i. Did authors find gender as a cofounder or important variable that is associated with Fever + (cough or shortness of breath)/Any symptom? Please clarify this part. In other words, I would like to know the reason of stratification of age by gender.
      ii. At least for COVID Near You, there is enough samples for other/no response group. Please modify Table 3 and add that group result to the Table.
      iii. Why not to consider age as an individual variable without being classified by gender and be added to the Tables. How about adding gender (F/M/other) to the Tables as well?
      iv. Tables 2 and 3 can’t be followed easily. Please modify the tables.
      v. (Rao-Scott) Chi-squared/Fisher tests assess the association between two categorical variables, or comparing proportions across cells for a given variable. Authors considered these methods to compare the proportions of cells (e.g., age groups) for a given variable (e.g., any symptom), is it right?
      vi. Is there any reported testing results for Angus Reid Poll study (Table 2)?
   c. Why authors didn’t include Table for Forum and mainstreet research phone poll? Please clarify this part.
2. I suggest authors consider parametric methods (e.g., logistic regression model) to add more results regarding the association between the demographic variables and two main variables (1) Fever + (cough OR shortness of breath) vs other symptom and (2) Reported positive test result/not.
3. In addition to the previous comment, how about comparing the results between web and phone-based sources? Authors introduced these three data sources, however there is not enough results to compare the data across these three studies.
4. Authors should be more precise about calling Tables across manuscript. Page 8, line 1 (results related to the COVID Near You), shows Table 3, not Table 2.
5. To improve results due to the lack of samples, integrating these three studies using meta-analysis approaches may improve results and power of analysis.
