# Supplementary material for: Web and phone-based COVID-19 syndromic surveillance in Canada: A cross-sectional study
Source: PLoS One. 2020 Oct 2;15(10):e0239886. doi: 10.1371/journal.pone.0239886 (PMC7531838; doi:10.1371/journal.pone.0239886)
Supplement: Attachment — Submitted filename: Responses_ August192020.docx [file pone.0239886.s006.docx]

**AUTHOR RESPONSES**

**1. Please ensure that your manuscript meets PLOS ONE's style requirements, including those for file naming. The PLOS ONE style templates can be found at**

**https://journals.plos.org/plosone/s/file?id=wjVg/PLOSOne_formatting_sample_main_body.pdf and**

**https://journals.plos.org/plosone/s/file?id=ba62/PLOSOne_formatting_sample_title_authors_affiliations.pdf**

**2. In ethics statement in the manuscript and in the online submission form, please provide additional information about the patient records used in your retrospective study. Specifically, please ensure that you have discussed whether all data were fully anonymized before you accessed them and/or whether the IRB or ethics committee waived the requirement for informed consent. If patients provided informed written consent to have data from their medical records used in research, please include this information.**

Thank you, we have added this information to the manuscript (page 4).

**3. Thank you for including your competing interests statement; "I have read the journal's policy and the authors of this manuscript have the following competing interests: WJ Schatten is a paid employee for Forum Research. II Bogoch has consulted to BlueDot, a social benefit corporation that tracks the spread of emerging infectious diseases. The remaining authors have no disclosures."**

**We note that one or more of the authors are employed by a commercial company: Forum Research**

1. **Please provide an amended Funding Statement declaring this commercial affiliation, as well as a statement regarding the Role of Funders in your study. If the funding organization did not play a role in the study design, data collection and analysis, decision to publish, or preparation of the manuscript and only provided financial support in the form of authors' salaries and/or research materials, please review your statements relating to the author contributions, and ensure you have specifically and accurately indicated the role(s) that these authors had in your study. You can update author roles in the Author Contributions section of the online submission form.**

We have amended the Funding section at the end of the manuscript (page 20-21). Notably, Forum & Mainstreet Research had previously collected the data used in this study, as we had indicated in our first submission. The funding organization played no role in study design, collection of data from the other two sources, analysis, decision to publish or preparation of the manuscript. Of note, the corresponding author first developed the study concept and initiated the request for use of Forum & Mainstreet polling data.

**Please also include the following statement within your amended Funding Statement. “The funder provided support in the form of salaries for authors [insert relevant initials], but did not have any additional role in the study design, data collection and analysis, decision to publish, or preparation of the manuscript. The specific roles of these authors are articulated in the ‘author contributions’ section.” If your commercial affiliation did play a role in your study, please state and explain this role within your updated Funding Statement.**

We have done this (page 20-21). As described above, this study was undertaken using previously collected polling data shared by Forum & Mainstreet research with the study team (and at the corresponding author’s request).

**2. Please also provide an updated Competing Interests Statement declaring this commercial affiliation along with any other relevant declarations relating to employment, consultancy, patents, products in development, or marketed products, etc.**

**Within your Competing Interests Statement, please confirm that this commercial affiliation does not alter your adherence to all PLOS ONE policies on sharing data and materials by including the following statement: "This does not alter our adherence to  PLOS ONE policies on sharing data and materials.” (as detailed online in our guide for authors** [**http://journals.plos.org/plosone/s/competing-interests**](about:blank)**) . If this adherence statement is not accurate and  there are restrictions on sharing of data and/or materials, please state these. Please note that we cannot proceed with consideration of your article until this information has been declared.**

The commercial affiliation does not alter our adherence to PLOS ONE policies on sharing data and materials. However, as we stated in our initial submission, the data used in this study does not belong to the study team, but rather to the three different groups that shared it with the study team: Angus Reid Institute, Forum research, and COVID Near You. Requests for data access should go to info@angusreid.org, wschatten@forumresearch.ca, or john.brownstein@childrens.harvard.edu, respectively. Data from COVID Near You are available for public health surveillance and can be requested at: https://www.atscale.com/covidnearyou/

**Please include both an updated Funding Statement and Competing Interests Statement in your cover letter. We will change the online submission form on your behalf.**

We have done this, thank you.

**Please know it is PLOS ONE policy for corresponding authors to declare, on behalf of all authors, all potential competing interests for the purposes of transparency. PLOS defines a competing interest as anything that interferes with, or could reasonably be perceived as interfering with, the full and objective presentation, peer review, editorial decision-making, or publication of research or non-research articles submitted to one of the journals. Competing interests can be financial or non-financial, professional, or personal. Competing interests can arise in relationship to an organization or another person. Please follow this link to our website for more details on competing interests:** [**http://journals.plos.org/plosone/s/competing-interests**](about:blank)

**4. We note you have included a table to which you do not refer in the text of your manuscript. Please ensure that you refer to Table 3 in your text; if accepted, production will need this reference to link the reader to the Table.**

Thank you for pointing this out. This was a typographical error and we have corrected it.  **Additional Editor Comments (if provided):

I appreciate your paper, but need some revisions.
Following reviewer suggestions you can improve your manuscript**

Thank you very much for these.

**Reviewer #1: This article provides estimates of the prevalence of COVID-19 symptoms in Canada which is valuable for modelling and public health planning. A few points for consideration:
1) How are the Angus Reid Forum panel members selected? Is this a random sample of Canadians?**

Thank you for this question. Angus Reid Forum panel members (a group of over 50,000 Canadian residents) have volunteered to be regularly invited to fill out surveys in exchange for gift card rewards or entry into sweepstakes draws (more details at <https://www.angusreidforum.com/FAQ>). Respondents to this COVID-19 poll were randomly selected from within the Angus Reid Forum. This information (and reference) was added to the Methods section (p. 4).

**2) At the start of page 8, Table 2 is referenced and I believe this should be Table 3.**

Thank you for pointing this out. This was a typographical error and we have corrected it.  **3) Verify data in Table 3 as the proportion reporting at least one symptom for Ontario on page 8 differs from what is in Table 3**

Thank you for pointing this out. This was also a typographical error and we have corrected it- it should have read “Nova Scotia” in the text rather than “Ontario”.

**4) No regional differences were observed with the Angus Reid data but regional differences were observed in the COVID near you data. Any reasons to explain why?**

Great question, thank you for bringing this up. We have added this to the discussion (p. 11):

“Whereas there were significant interprovincial differences in the proportion of COVID Near You respondents with symptoms, this was not the case for the Angus Reid poll. This may reflect differences in sample size, where a greater number of responses to COVID Near You means that even small absolute differences in proportions reached statistical significance. Nonetheless, differences observed across provinces in both COVID Near You and the Angus Reid poll did not reflect confirmed COVID-19 case activity in these locations. For example, in COVID Near You, British Columbia and Nova Scotia had the highest proportion reporting at least one COVID-19 symptom. Yet, during March-April 2020, Quebec had considerably more cases than any other province.24 This likely reflects regional differences in survey uptake. Hence, some caution is warranted in attempting to compare rates of symptoms across provinces.”

**Reviewer #2: Title: Web and Phone-based COVID-19 Syndromic Surveillance in Canada: A Cross-Sectional Study**

**This paper presents a study to describe the characteristics, symptoms, and self-reported testing rates of respondents in three different COVID-19 symptom surveys in Canada. However, there are questions that limit my enthusiasm of the paper, as outlined below.**

1. **Results:**
   a. **Table 1: Authors considered (-) and 0. What I guess (-) shows cell<6. So please define that at the caption and fix that across all 3 tables. We don’t expect to have both 0 and (-) across tables.**

Thank you for pointing this out. The reason for doing is was to reduce the identifiability of individuals within small cells. However, we have now changed it such that all cells <6 including 0 are now all listed as “-“.

b. **Table 2: Authors did stratification for the age based on the gender.**
   i. **Did authors find gender as a cofounder or important variable that is associated with Fever + (cough or shortness of breath)/Any symptom? Please clarify this part. In other words, I would like to know the reason of stratification of age by gender.**

The reason we did this is because we know that females and males may experience the effects age differently. In general, the study team has experienced a push to stratify results by gender, as encouraged by Canada’s major public research funder (but not the funder of this study), the Canadian Institutes of Health Research (CIHR). However, we are happy to present these separately. We have done this the revised Tables 2 and 3, and the related text in Results.

ii. **At least for COVID Near You, there is enough samples for other/no response group. Please modify Table 3 and add that group result to the Table.**

We have done this thank you.

iii. **Why not to consider age as an individual variable without being classified by gender and be added to the Tables. How about adding gender (F/M/other) to the Tables as well?**

We have done this, thank you for the suggestion.

iv. **Tables 2 and 3 can’t be followed easily. Please modify the tables.**

We have added details to the table footnotes to enhance clarity and ease of interpretation.

v. **(Rao-Scott) Chi-squared/Fisher tests assess the association between two categorical variables, or comparing proportions across cells for a given variable. Authors considered these methods to compare the proportions of cells (e.g., age groups) for a given variable (e.g., any symptom), is it right?**

Yes exactly. For age group by any symptom, the p-value reflects the p-value for the Chi-square of any symptom (yes/no) by age group. Clarifying information has been added to the footnotes of tables 2-4.

vi. **Is there any reported testing results for Angus Reid Poll study (Table 2)?** Unfortunately there were no questions about test results in the Angus Reid poll.
c. **Why authors didn’t include Table for Forum and mainstreet research phone poll? Please clarify this part.**

We have added a small table for these results (Table 4). Because the unit of reporting in this poll was the household, we have only included results within household groupings (household income and household size).

2. **I suggest authors consider parametric methods (e.g., logistic regression model) to add more results regarding the association between the demographic variables and two main variables (1) Fever + (cough OR shortness of breath) vs other symptom and (2) Reported positive test result/not.**

We appreciate this suggestion, and indeed we had previously considered including a multivariable model, but opted against it since it did not suit our overall study aim. The purpose of this study was entirely descriptive, to describe the characteristics of Canadian residents reporting COVID-19 symptoms, testing, and, in some cases, positive test results. We did not seek to identify independent predictors of having symptoms or of test positivity.

3. **In addition to the previous comment, how about comparing the results between web and phone-based sources? Authors introduced these three data sources, however there is not enough results to compare the data across these three studies.**

There is considerable heterogeneity across these studies. Given this variability in data collection and result reporting, we opted to qualitatively compare the studies and their results, rather than test for statistically significant difference between data sources. Such differences would be expected to be significant, and their meaning would have been extremely challenging to meaningfully interpret given divergent time periods, populations, methodology, and questions.

4. **Authors should be more precise about calling Tables across manuscript. Page 8, line 1 (results related to the COVID Near You), shows Table 3, not Table 2.**

Thank you for pointing this out. This was a typographical error and we have corrected it.

5. **To improve results due to the lack of samples, integrating these three studies using meta-analysis approaches may improve results and power of analysis.**

We completely agree that a meta-analytic approaches would be appropriate for related studies, but in this manuscript there is marked heterogeneity between studies in terms of methods. We believe that combining estimates across data sources would provide the impression that they are directly comparable, which they are not. Rather, their findings can be qualitatively combined to provide a picture of low test performance rates among symptomatic persons in Canada in March and April 2020.
